# Dietary Modulation of Oxidative Stress From Edible Insects: A Mini-Review

**DOI:** 10.3389/fnut.2021.642551

**Published:** 2021-02-26

**Authors:** Veronica D'Antonio, Mauro Serafini, Natalia Battista

**Affiliations:** Faculty of Biosciences and Technologies for Agriculture, Food and Environment, University of Teramo, Teramo, Italy

**Keywords:** entomophagy, antioxidants, edible insects, novel foods, functional foods, oxidative stress, sustainable nutrition

## Abstract

Edible insects are proposed as a nutritious and environmentally sustainable alternative source to animal proteins, due to their numerous advantages in terms of reduced ecological impact and high nutritional value. However, the novelty for edible insects relies on the content of bioactive ingredients potentially able to induce a functional effect in the body. The present review summarizes the main findings on the antioxidant properties of edible insects available in the literature. A total of 30 studies involving animals, cell cultures, or *in vitro* experimental studies evaluating the antioxidant effect of edible insects are presented in this work. When the antioxidant activity was investigated, using a wide variety of *in vitro* tests and in cellular models, positive results were shown. Dietary supplementation with edible insects was also able to counteract dietary oxidative stress in animal models, restoring the balance of antioxidant enzymes and reducing the formation of oxidation damage markers. On the basis of the reviewed evidences, edible insects might represent a source of novel redox ingredients at low ecological impact able to modulate oxidative stress. However, due to the fact that majority of these evidences have been obtained *in vitro* and in cellular and animal models, dietary intervention trials are needed to assess the efficacy of edible insect consumption to modulate redox status in humans.

## Introduction

Entomophagy, the practice of eating insects and invertebrates, has been part of human history for centuries, playing a significant role in cultural and religious practices. Recently, a new global interest in edible insects and invertebrates arises from the impellent necessity of preserving agriculture resources to feed the 9 billion world's population predicted for 2050 and to obtain a drastic reduction of the ecological impact of food production, accounting for between 20 and 30% of the total environmental impact (1). In terms of ecological impact, edible insects are characterized by a negligible greenhouse gas (GHG) emission as well as water and ecological footprints, meeting the population's need for a more sustainable protein supply. In this view, Onnincx et al. (2) have described lower NH_3_ emission level, higher average daily gain, and a comparable or lower CO_2_ (g/kg mass gain) production of insects, which result in a higher feed conversion efficiency, with respect to conventional livestock. Moreover, although the fossil energy needed to mealworms rearing is comparable to or higher than conventional food sources such as milk or different meats, these insects produce reduced GHG—one of the main factors inducing climate changes—and the space required for their rearing is much lower than conventional livestock (3).

Edible insects are characterized by a high nutritional value, and they are good potential sources of proteins, amino acids, minerals, and lipids (4). The protein content of the various species of insects is generally very high (50–70% on dry basis), while lipids represent the second largest fraction of the nutritional composition, ranging from 10 to 50% on dry basis and depending on life stage (higher in the larval stage) and species. Interestingly, it has been reported that they have a higher value of the essential fatty acids linoleic (18:2 *n*-6) and α-linolenic (18:3 *n*-3) when compared to other conventional sources (4). However, the innovative feature of edible insects relies on the content of bioactive ingredients and on their ability to induce a functional effect in the body and potentially able to provide a protective effect against diseases, entomotherapy, as previously suggested (5). In the last years, scientific evidences on the functional properties of edible insects have been provided in different experimental models, with majority of the studies focused on the understanding of the antioxidant role of edible insects and their extracts (6, 7). In order to understand if edible insects might play a role in the dietary modulation of oxidative stress, in this mini-review, we aim to summarize the available evidences regarding the antioxidant role of edible insects and invertebrates in *in vitro, ex vivo*, and *in vivo* models.

## Search Strategy

A search for literature investigating the antioxidant effect of edible insects was carried out by PubMed database (National Library of Medicine, Bethesda, MD) using the following keywords: “edible insect,” “oxidative,” and “antioxidant.” Eligible studies for this mini-review have included randomized controlled trials in humans, experimental animals, or cell cultures or *in vitro* experimental studies published in English, leading to the selection of 30 studies. No studies on human subjects were available.

## Results

### *In vitro* Antioxidant Activities of Edible Insects

Table 1 (6–24) shows the studies investigating the *in vitro* antioxidant activity of edible insects and invertebrate fractions. Several methods were taken into account: the most used was the 1,1-diphenyl-2-picrylhydrazyl (DPPH), performed in 14 papers (7–15, 18–20, 23, 24), while 2,2′-azino-bis(3-ethylbenzothiazoline-6-sulfonic acid) (ABTS) and ferric reducing antioxidant power (FRAP) were utilized in 11 (6, 7, 10, 11, 13, 14, 16, 17, 19, 20, 22) and 8 studies (6, 10–12, 14, 15, 19, 21), respectively. Antioxidant activity was investigated also as scavenging activity against different radicals, metal ion chelating activity, reducing power, or, only for the paper of Sun et al. (25), with β-carotene and linolenic acid bleaching tests. Nineteen studies were carried out, involving 30 species of insects; of these, the most studied was *Tenebrio molitor*, cited in nine papers (6–10, 13–16), followed by *Acheta domesticus* (6, 8, 9) and *Gryllodes sigillatus* (7, 19, 20), tested in three papers, and *Bombyx mori* (6, 11), *Hermetia illucens* (21, 22), and *Lethocerus indicus* (6, 24) in two papers. Water-soluble fractions were tested in 12 papers (6, 9–15, 17, 18, 20, 24), protein hydrolysates were evaluated in 6 research articles (7, 8, 16, 19, 21, 22), while lipo-soluble fractions were reported in 2 papers only (6, 23). Interestingly, all tested fractions showed a significant antioxidant activity with the only exception of the cricket *Gryllus sigillatus* protein hydrolysates that did not show any positive results using the FRAP method, while an antioxidant capacity was recorded using ABTS, DPPH, and metal ion chelating activity methods (19). The study conducted by Di Mattia et al. (6) was the only one providing a comparison between the antioxidant activity of water- and lipo-soluble fraction edible insects and foods like fresh orange juice and olive oil. Results showed that water-soluble extracts of grasshoppers, silkworm, and crickets display the highest values of antioxidant capacity, expressed as Trolox Equivalent Antioxidant Capacity (TEAC), 5-fold higher than fresh orange juice. Furthermore, water-soluble extracts of grasshoppers, African caterpillars, and crickets had a reducing power (FRAP) double than that of fresh orange juice. As regards the lipo-soluble fraction, silkworm, evening cicada, and African caterpillars showed a TEAC twice than that of olive oil. Differently from other studies, Dutta et al. (18) showed that aqueous extract of *Vespa affinis* was able to increase the activity of the endogenous antioxidant enzymes catalase (CAT) and glutathione S-transferase (GST) (18).

**Table 1:** *In vitro* antioxidant activity of edible insects and invertebrates.

**Edible insects - extract**	**Concentrations**	**Antioxidant method**	**Result**	**References**
*Acheta domesticus, Tenebrio molitor*- PH	0.05–5 mg/ml	DPPH	+	Messina et al. (8)
*Acheta domesticus, Tenebrio molitor*- WS	10 mg/ml	DPPH	+	Navarro del Hierro et al. (9)
*Allomyrina dichotoma, Apis mellifera, Gryllus bimaculatus, Protaetia brevitarsis,Teleogryllus emma, Tenebrio molitor*- WS	500 μg/ml	ABTS, DPPH, FRAP	+	Pyo et al. (10)
	200 μg/ml	Nitric scavenging activity	+	
*Bombyx mori*- WS	10 g/50 ml	DPPH, ABTS, FRAP	+	Anuduang et al. (11)
*Brachytrupes orientalis*- WS	0.25–6.25 mg/ml	DPPH	+	Dutta et al. (12)
	1.25–12.5 mg/ml	FRAP, SAHR, SRSC	+	
*Tenebrio molitor*- WS	–	ABTS, DPPH	+	Son et al. (13)
*Tenebrio molitor*- WS	3 g/10 ml	ABTS, DPPH, FRAP	+	Mancini et al. (14)
*Tenebrio molitor*- WS	0.625–5.0 mg/ml	DPPH, FRAP, ORAC, SAHR, hydrogen peroxide radical scavenging activity	+	Tang et al. (15)
*Tenebrio molitor, Ulomoides dermestoides*- PH	0.1–1.0 mg/ml	ABTS	+	Flores et al. (16)
*Pachymerus nucleorum*- WS	1 g/100 ml	ABTS	+	Alves et al. (17)
*Vespa affinis* L. - WS	0.25–6.25 μg/μl	DPPH	+	Dutta et al. (18)
	1.25–15 μg/μl	SAHR, SRSC	+	
	1.25–10 μg/μl	Activities of CAT and GST enzymes	+	
*Gryllodes sigillatus*- PH	1 mg/ml	ABTS, DPPH, metal ion chelating activity	+	Hall et al. (19)
		FRAP	-	
*Gryllodes sigillatus, Schistocerca gregaria, Tenebrio molitor*- PH	–	ABTS, DPPH, Fe^2+^ chelating activity	+	Zielinska et al. (7)
*Gryllus bimaculatus*- WS	–	ABTS	+	Hwang et al. (20)
	-	DPPH	+	
*Hermetia illucens*- PH	14 g/l	SAHR	+	Mintah et al. (21)
*Hermetia illucens*- PH	2 mg/ml	ABTS	+	Mintah et al. (22)
	4 mg/ml	FRAP, SRSC	+	
*Clanis bilineata*- LS	10–200 μg/ml	DPPH	+	Sun et al. (23)
	0.1–4 mg/ml	β-carotene and linolenic acid bleaching test	+	
Various (*Acheta domesticus, Alphitobius diaperinus, Bombyx mori, Calliptamus italicus, Imbrasia oyemensis, Lasius niger, Lethocerus indicus, Rhynchophorus ferrugineus, Scolopendra, Tanna japonensis, Tenebrio molitor, Haplopelma albostriatum, Pandinus imperator -* WS and LS)	–	ABTS hydro, ABTS lipo, FRAP	+	Di Mattia et al. (6)
Various (*Crocothemis servilia, Cybister tripunctatus, Hydrophilus olivaceous, Laccotrephes maculatus, Lethocerus indicus*- WS)	1–500 mg/ml	DPPH	+	Shantibala et al. (24)

*ABTS, 2,2′-azino-bis(3-ethylbenzothiazoline-6-sulfonic acid); CAT, catalase; DPPH, 2,2-diphenyl-1-picrylhydrazyl; FRAP, ferric reducing antioxidant power; GST, glutathione S-transferase; LS, lipo-soluble extracts; ORAC, Oxygen radical absorbance capacity; PH, protein hydrolysates; SAHR, scavenging activity on hydroxyl radicals; SRSC, superoxide radical scavenging capacity; WS, water-soluble extract*.

### Antioxidant Activity of Edible Insects in Cells and Animal Models

Table 2 summarizes the results obtained on the antioxidant activity of different insects and invertebrates; eight were tested in cellular models (12, 13, 18, 20, 26–29) and as many in animal models (20, 27, 30–35). For what concerns cellular models, the effects of the water-soluble extract of dung beetles of *Onitis* sp., mole crickets of *Gryllotalpa* sp., grasshopper of *Caelifera* sp. (28), *Oryctes boas*, and *Zonocerus variegatus* (29) on the oxidative status of human peripheral blood lymphocytes were evaluated, showing that, at lower concentrations of all of their WEs, they display antioxidant activities, but at higher concentrations, their effects switched to prooxidant. Interestingly, intermediate concentrations did not affect the antioxidant efficiency. According to the results obtained in a cell-free system (18), the aqueous extract of *V. affinis* was able to increase the activity of both GST and CAT and to reduce reactive oxygen species (ROS) also in THP-1 human monocytes and human plasma. Oxidative stress induced by high glucose treatment caused a significant decrease of both Nrf2 and GST protein expression in C2C12, a murine myotubes cell line, and that the supplementation with the hydro-alcoholic extract of *Brachytrupes orientalis* re-established the normal levels of both proteins and prevented the high glucose-induced oxidative impairment in terms of ROS and malondialdehyde (MDA) levels (12). Three different studies have underlined the capacity of aqueous extracts of *Gryllus bimaculatus* (20), the methanolic extract of defatted powder and usaponifiable lipids, obtained by *T. molitor* (13), and *B. mori* protein hydrolysates to reduce nitric oxide (NO) production in lipopolysaccharide-induced RAW 264.7, a murine macrophage cell line. NO production was reduced also in D-HMVECs, i.e., diabetic type 2 microvascular endothelial cells, by glycosaminoglycan from *G. bimaculatus* (27). In agreement with the paper of Yoon and coworkers (26), protein hydrolysates of *T. molitor* and *G. bimaculatus* did not exert any effect on NO release.

Table 2Antioxidant activity of edible insects and invertebrates in cellular and animal models.

**Table:** 

**Edible insects - extract**	**Cell type**	**Dose**	**Antioxidant/Oxidant markers**	**References**
**Cellular studies**				
*Bombyx mori*- PH	RAW264.7	0.1, 0.3, 0.5 mg/ml	NO	Yoon et al. (26)
*Gryllus bimaculatus*- PH			NO ↔	
*Tenebrio molitor*- PH			NO ↔	
*Brachytrupes orientalis*- WS	C2C12	7.5, 10*, 12.5* mg/ml	Lipid peroxidation: MDA ↓^*^; ROS ↓^*^; GST ↑^*^	Dutta et al. (12)
*Gryllus bimaculatus - CP*	D-HMVECs	5, 10 mg/ml	NO ↓	Ahn et al. (27)
*Gryllus bimaculatus*- WS	RAW264.7	20–100 μg/ml	NO ↓	Hwang et al. (20)
*Onitis* sp. - WS	hPBL	5–2,000 ppm	TOS ↔ (↑ 2,000 ppm) TAC ↔↑15 ppm, ↓1,000, 2,000 ppm	Koc et al. (28)
*Gryllotalpa* sp. - WS			TOS ↔ (↑ 2,000 ppm); TAC ↔ (↑10 ppm, ↓1,000, 2,000 ppm)	
*Caelifera* sp. - WS			TOS ↔ (↑ 2,000 ppm); TAC ↔ (↓ 2,000 ppm)	
*Oryctes boas*- WS	hPBL	5–2,000 ppm	TAC ↑ (10–40 ppm), ↓ (2,000 ppm) TOS ↑ (1,000, 2,000 ppm)	Memis et al. (29)
*Zonocerus variegatus*- WS			TAC ↑ (10–25 ppm), ↓ (500–2,000 ppm) TOS ↑ (200–2,000 ppm)	
*Tenebrio molitor*- WS, LS	RAW264.7	WS: 25–500 μg/ml LS: 0.05–5 μg/ml	NO ↓	Son et al. (13)
*Vespa affinis*- WS	THP-1	0.4, 0.8*, 1.2*μg/μl	GST, CAT ↑	Dutta et al. (18)
	THP-1	0.8 μg/μl	ROS ↓	
	hPlasma	1.25–10.00 μg/μl	GST ↑ (except for 1.25 and 2.50 μg/μl) CAT ↑ (except for 1.25 μg/μl)

**Table:** 

**Animal studies**					
**Edible insects - extract**	**Animal/Disease**	**Dose**	**Treatment duration**	**Antioxidant/Oxidant markers**	**References**
*Bombyx mori*- LS	Wistar rats, hypercholesterolemia	1, 2*, 4** ml/kg/day	6 weeks	Serum: TAC*, SOD *, **, GPx *,**↑; MDA ↓ Liver: TAC, SOD ↑; GPx ↔; MDA ↓	Zou et al. (30)
Green cocoon shell of *Bombyx mori*- WS	ICR mice, type 2 diabetes	150, 250*, 350* mg/kg	7 weeks	Liver: GPx, SOD ↑; MDA, 8-OHdG ↓^*^	Zhao et al. (31)
*Gryllus bimaculatus*- WS	Wistar rats, obesity	100, 200* mg/kg	2 months	Blood protein carbonyl content ↓^*^(2 m) CAT ↔ Liver: MDA↓ Serum: uric acid ↔↓(1 m), ↑(2 m)	Ahn et al. (32)
*Gryllus bimaculatus - CPs*	BKS.Cg-m+/+Leprdb mice, diabetes	5 mg/kg	1 month	Carbonyl content: blood ↓, liver ↔. GST ↔; CAT, GPx ↑	Ahn et al. (27)
*Gryllus bimaculatus - WI*	Sprague–Dawley rats, varicocele	1.63, 6.5 mg/kg	42 days	Testicular tissues:MDA, ROS/RNS ↓SOD, GPx, CAT ↑	Karna et al. (33)
*Gryllus bimaculatus*- WS	C57BL/6J mice, alcoholic liver damage	200 mg/kg	2 weeks	Liver: 8-OHdG, MDA ↓ Small intestine: 8-OHdG ↓	Hwang et al. (20)
*Protaetia brevitarsis*- WS	C57BL/6 mice, obesity	100, 200* mg/kg/day	7 weeks	Liver: GPx ↑; CAT ↑^*^	Ahn et al. (34)
*Tenebrio molitor*- WS (fermented)	Sprague–Dawley rats, alcoholic liver disease	50, 100, 200 mg/kg/day	8 weeks	Liver: β-oxidation ↑	Choi et al. (35)

*8-OHdG, 8-hydroxy-2′ deoxyguanosine; CAT, catalase; CP, compound; Gpx, glutathione peroxidase; GST, glutathione S-transferase; LS, lipo-soluble extracts; MDA, malondialdehyde; NO, nitric oxide; PH, protein hydrolysates; RNS: reactive nitrogen species; ROS, reactive oxygen species; SOD, superoxide dismutase; TAC, total antioxidant capacity; TOS, total oxidant status; WI, whole insect; WS, water-soluble extract*.

A total of eight intervention studies on animal models characterized by hypercholesterolemia, diabetes, obesity, and alcoholic liver damage have been published. In more detail, Zou et al. (30) reported that in Wistar rats with hypercholesterolemia, *B. mori* pupae oil supplementation was able to restore superoxide dismutase (SOD) levels, increasing total antioxidant capacity (TAC) levels, reducing MDA in liver and serum, and restoring the activity of glutathione peroxidase (GPx) in rats' liver, stressed by a high-cholesterol diet. Moreover, the supplementation with ethanol extract of the sericin layer from the green cocoon shell of *B. mori* increased liver GPx and SOD in obese mice with type 2 diabetes, and the treatment also reduced the liver content of MDA and 8-hydroxy-2′-deoxyguanosine (8-OHdG), as markers of lipid and DNA oxidative damage (31). Ethanolic extract of *G. bimaculatus*, added to a high-fat diet, did not affect serum CAT in obese rats (32). However, the prolonged treatment with ethanolic extract of *G. bimaculatus* repaired the protein and lipid oxidative damage caused by high-fat diet in both liver and blood, where serum uric acid—the final oxidation product of purine metabolism—and carbonyl—a marker of protein oxidation—concentrations were reduced (32). In a study carried out in 2019 by Hwang et al. (20), they reported the positive action of the aqueous extract of *G. bimaculatus* in restoring the normal physiological levels of 8-OHdG levels and MDA content in liver and small intestine of C57BL/6J mice liver damage caused by acute alcohol exposure. Moreover, treatment with *G. bimaculatus* significantly restored the increased levels of MDA, ROS, and reactive nitrogen species (RNS) and the reduced levels of SOD, GPx, and CAT in testicular tissue of Sprague–Dawley rats affected by varicocele (33). Glycosaminoglycan extracted from *G. bimaculatus* and administered for 1 month to BKS.Cg-m+/+Lepr^db^ diabetic mice reduced blood carbonyl content, but not that of liver. It also did not affect GST, but improved CAT and GPx levels (27). The ethanolic extracts of *Protaetia brevitaris* larvae, administered with a high-fat diet for 7 weeks, increased GPx and CAT in liver of obese C57BL/6J mice (34). Finally, the treatment fermented defatted *T. molitor* powder of Sprague–Dawley rats fed with a chronic alcohol diet dose-dependently increased hepatic β-oxidation (35).

## Discussion

In this review, we showed that different species of edible insects display an antioxidant activity in *in vitro* models and are able to modulate induced oxidative stress in cellular and animal models. All the insects tested *in vitro* and in different cellular models, except one, display radical scavenging or metal ion chelation properties, as well as modulation of antioxidant enzymes. Results in animal models have clearly shown, in all the studies, that the increased content of markers of oxidative damage markers, induced by the dietary stress, was reversed following the treatment with edible insects, restoring the impaired activity of antioxidant enzymes, and by reducing oxidation products. Redox status was evaluated through the ability of the insect extracts to reduce ROS (three studies) or to increase total antioxidant status (two studies) in cellular models. Moreover, specific oxidation markers were chosen: the urinary excretion of 8-OHdG, a predictive risk factor for cancer, atherosclerosis, and diabetes (36), and MDA that plays a critical role in atherosclerosis by its capacity to drive inflammatory processes (37). These oxidation products were studied respectively in four *in vivo* interventions, one in cells for MDA and in two *in vivo* interventions for 8-OhdG, also in those cases with positive results. One intervention (32) has also evaluated the content of serum uric acid—the final oxidation product of purine metabolism—and two interventions (27, 32) have evaluated that of carbonyl—a marker of protein oxidation. In this context, we should recall that protein carbonyl levels are elevated in several pathological conditions, including neurodegenerative diseases, obesity, or diabetes (38); on the other hand, serum uric acid levels can be a marker of renal and cardiovascular risk, in particular as a consequence of diabetes (39). The antioxidant enzymes that were taken into account in these studies were CAT, which was studied in six different interventions (four *in vivo*, one in a cellular model, and one in a cell-free system); GPx, which was investigated in five *in vivo* studies; SOD, which was analyzed in three *in vivo* studies; and GSTs, which were evaluated in one *in vivo* intervention, two interventions in cellular models, and one *in vitro*. Moreover, Nrf-2—a transcription factor that acts as a master regulator of the antioxidant response system and whose activity declines with age as well as with degenerative disorders (38)—was reported to increase only in one study carried out in a myotube cell line. Conflicting results arose from human studies that evaluated the relationship between diseases or aging and antioxidant enzymes: indeed, an increase in antioxidant enzymes can also be related to a high response to oxidation (38). However, in all the examined studies, the oxidative stress induced a reduction of these enzyme levels that was prevented by insect supplementation. Based on these evidences, it can be stated that, in the applied conditions, edible insects exert mostly a positive effect on the modulation of antioxidant enzymes.

On the basis of the positive results of the three studies focused on NO production in macrophages, radical involved in the modulation of inflammation and immune regulation (40), it might be speculated that edible insect extracts might also have a potential anti-inflammatory activity due to their ability to reduce the release of NO.

As regards the insects, 35 different species were investigated; the ones that arouse major interest in researchers were *T. molitor* and those belonging to the Gryllydae family, respectively studied in 13 and 10 different researches. However, it is interesting to note that the experimental studies carried out *in vivo* are mainly focused on *G. bimaculatus, P. brevitaris*, and *B. mori*, while curiously, all the studies performed using *T. molitor* are *in vitro*, with only one exception (35).

## Conclusions

On the basis of the reviewed evidences, edible insects might represent a source of novel redox ingredients at low ecological impact able to modulate oxidative stress. However, due to the fact that majority of these evidences have been obtained *in vitro* and in animal models, dietary intervention trials are needed to confirm the antioxidant efficacy of edible insects in humans.

## Author Contributions

MS conceived the review topics. VD'A wrote the initial draft. MS and NB revised and supervised the entire work. All authors approved the submitted version.

## Conflict of Interest

The authors declare that the research was conducted in the absence of any commercial or financial relationships that could be construed as a potential conflict of interest.

## Abbreviations

8-OHdG: 8-hydroxy-2′-deoxyguanosine
ABTS, 2: 2′-azino-bis(3-ethylbenzothiazoline-6-sulfonic acid)
CAT: catalase
DPPH, 1: 1-diphenyl-2-picrylhydrazyl
CP: compound
FRAP: ferric reducing antioxidant power
GHG: greenhouse gas emissions
GPx: glutathione peroxidase
GST: glutathione S-transferase
LS: lipo-soluble extract
MDA: malondialdehyde
NO: nitric oxide
Nrf2: Nuclear factor erythroid 2-related factor
ORAC: oxygen radical absorbance capacity
PH: protein hydrolysates
RNS: reactive nitrogen species
ROS: reactive oxygen species
SAHR: scavenging activity on hydroxyl radicals
SOD: superoxide dismutase
SRSC: superoxide radical scavenging capacity
TAC: total antioxidant capacity
TEAC: Trolox equivalent antioxidant capacity
TOS: total oxidant status
WI: whole insect
WS: water-soluble extract.
